# Magnetic molecules lose identity when connected to different combinations of magnetic metal electrodes in MTJ-based molecular spintronics devices (MTJMSD)

**DOI:** 10.1038/s41598-023-42731-9

**Published:** 2023-09-27

**Authors:** Eva Mutunga, Christopher D’Angelo, Pawan Tyagi

**Affiliations:** Mechanical Engineering, Center for Nanotechnology Research and Education (CNRE), University of the District of Columbia, 4200 Connecticut Ave. NW, Washington, DC 20008 USA

**Keywords:** Metamaterials, Organic-inorganic nanostructures

## Abstract

Understanding the magnetic molecules’ interaction with different combinations of metal electrodes is vital to advancing the molecular spintronics field. This paper describes experimental and theoretical understanding showing how paramagnetic single-molecule magnet (SMM) catalyzes long-range effects on metal electrodes and, in that process, loses its basic magnetic properties. For the first time, our Monte Carlo simulations, verified for consistency with regards to experimental studies, discuss the properties of the whole device and a generic paramagnetic molecule analog (GPMA) connected to the combinations of ferromagnet-ferromagnet, ferromagnet-paramagnet, and ferromagnet-antiferromagnet metal electrodes. We studied the magnetic moment vs. magnetic field of GPMA exchange coupled between two metal electrodes along the exposed side edge of cross junction-shaped magnetic tunnel junction (MTJ). We also studied GPMA-metal electrode interfaces’ magnetic moment vs. magnetic field response. We have also found that the MTJ dimension impacted the molecule response. This study suggests that SMM spin at the MTJ exposed sides offers a unique and high-yield method of connecting molecules to virtually endless magnetic and nonmagnetic electrodes and observing unprecedented phenomena in the molecular spintronics field.

## Introduction

The molecular spintronics field involves molecules with a wide range of quantum states^1^ and conductivity^2^ to yield futuristic memory devices and logic devices for regular computers to quantum computers^3–5^. Molecules are also the smallest designable and controllable nanoscale device elements mass-produced with single-atom precision and exotic quantum properties^6,7^. Molecules have been a strong candidate for revolutionizing computer technologies since 1950 and are now touted as highly promising candidates for quantum computers^8–11^. However, the advancement of molecular spintronics has been extremely slow due to the unsurmountable device fabrication challenges^12,13^. The capabilities of molecules are also underestimated due to the general perceptions about molecules’ properties based on studies on isolated molecules when they are not connected to metal electrodes^14^. Irrespective of device architecture type, understanding the interaction between different types of electrodes and magnetic molecules is critically important to attain knowledge for designing futuristic molecular spintronics devices(MSDs).

Density functional theory (DFT) has been the method of choice for studying molecular devices. DFT capabilities are further enhanced by the non-equilibrium green function (NEGF) to study the charge and spin transport through nanostructures connected to the metallic leads. DFT + NEGF are capable of dealing with devices comprising several hundred atoms when employing LCAO basis sets^15^. However, studying single-molecule magnet (SMM) based devices involving strong metal–molecule interaction is very challenging; it is noteworthy that there exists an immense complexity in simulating SMM alone. For the DFT study of paramagnetic molecules alone, important parameters were taken from the experimental studies to improve the accuracy of simulations^16^. Unfortunately, the experimental studies focusing on measuring SMM bridged between various magnetic electrodes via various functional groups are extremely limited. Hence, there is a lack of reference to guide the DFT studies or evaluate DFT results. As a promising development, the DFT accuracy has been further improved in the context of molecules connected to gold electrodes via different functional groups^17^. Different forms of first principle studies are experimented with to address self-interaction corrections at the gold metal–molecule interface in the context of molecular electronics devices^17^. DFT studies have been applied to investigate molecule spintronics devices involving paramagnetic molecules and nanoscale ferromagnetic electrodes^18^, analogous to nano-gap break junction devices. However, according to the best of our knowledge, DFT was not applied for validating the experimental studies on the macroscopic magnetic tunnel junctions (MTJ) and MTJ-based MSD, as discussed in this paper. However, a useful development has occurred in simulating MTJ that utilizes micromagnetic simulation for computing dynamic magnetic and transport properties^19^. However, the direct application of DFT and micromagnetic simulation is still very challenging in several experimentally observed phenomena on SMM and MTJ-based MSD (MTJMSD).

MTJMSD research targets the actual applications of MSD in computers and other new fields due to its robustness and mass-manufacturability^12^. It has been a daunting, unsolved task to produce the most popular break-junction-like device fabrication techniques with > 10% yield^20^. To the best of our knowledge, no conventional MSD fabrication approach could directly connect a molecule, not the several monolayers, to the dissimilar multilayer magnetic electrodes in a mass-producible manner^12^. To overcome this technology gap and address fabrication challenges, we have been developing MTJMSD for > 18 years^21–23^. In the MTJMSD strategy, almost any combination of metal electrodes can be robustly separated by an insulator, and almost any type of molecule with a suitable anchoring group can be bridged between the two metal electrodes along the exposed sides^22,23^. MTJMSDs have exhibited a large number of intriguing phenomena such as room temperature current suppression^24^, spin photovoltaic^25,26^,  > four orders of magnitude tunneling magnetoresistance changes^27^, and long-range molecule-induced magnetic ordering^28^. We have observed indications of molecule-induced unprecedented spin polarization on MTJMSD’s electrodes^29^; such indications of high spin polarization have also been observed elsewhere due to complete spin filtering in atomic-scale nickel oxides^30^. Spin filtering caused by the symmetry aspect of molecule-ferromagnetic electrode nano junction has been projected to yield a very high on/off resistance ratio^31,32^. Interestingly, spin filtering has been experimentally tested to yield more than 90% spin polarization^33,34^, surpassing the traditionally considered 50–70% spin polarization in conventional ferromagnetic electrodes used in MTJs^35^. Hence, the MSD design process must view SMM properties in conjunction with the strength of coupling with the magnetic electrodes.

Prior DFT studies have produced limited correlation with the experimentally observed phenomenon on our microscopic MTJMSD. DFT is undoubtedly useful in gaining insights about atomic orbital level interaction between molecules and different metal electrodes, for example, molecule-induced magnetic anisotropy^36^, but is of limited use in the context of MTJMSD. To address some of the simulation challenges, we have developed an alternative Monte Carlo simulation (MCS) route to provide insights into some experimentally observed phenomena on MTJMSDs. Our previous MCS study was accomplished on the molecule analog and pillar form MTJMSD^29^ to overcome the challenges of dealing with molecular-specific atomic-scale properties and molecule-ferromagnetic interactions. Our MCS approach is designed to connect the generic experimentally validated physics on related analogous nanoscale device systems and apply them to understand experimental observation on molecular devices; we were specifically encouraged by the prior work that applies the generic Simmons quantum tunneling model equation^37^ as well as quantum dot and single electron transistor device equations to understand the experimental results from molecular electronics devices without delving into molecule specific chemistry^38^. We were encouraged to see that our MCS approach accurately estimated the molecule coupling strength and provided valuable mechanism-related insights for some experimental observations^29^. Since then, we have utilized MCS studies to parametrically explore the impact of defects in the insulator on MTJMSD^39^, anisotropy^40^, and the strength of molecule-FM coupling on MTJMSD with different lengths and widths^41^. However, we did not explore the magnetic hysteresis response of MTJMSD under the impact of important factors such as (i) metal electrode types and (ii) MTJMSD geometry. These studies are necessary for making practical spintronics devices. This paper studies the magnetic moment vs. magnetic field of magnetic molecule state, molecule–metal interfaces, and complete MTJMSD.

## Methodology

**Figure 1:**
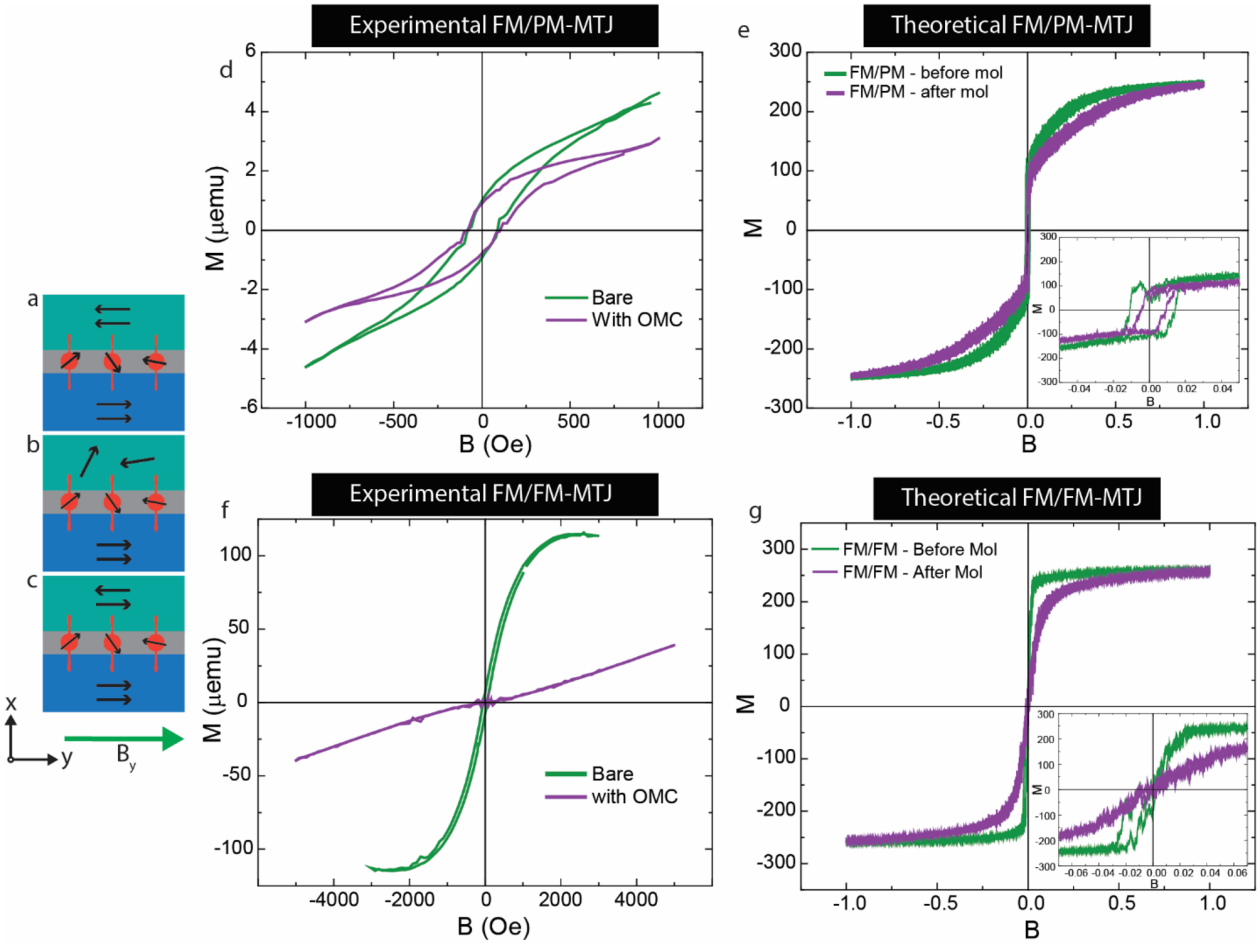
Schematic of a magnetic tunnel junction molecular spintronic device (MTJMSD) with paramagnetic molecules attached to: (**a**) two ferromagnetic electrodes, (**b**) a ferromagnetic and paramagnetic electrode, and (**c**) ferromagnetic and antiferromagnetic electrode. An in-plane external magnetic field is applied in the y-direction. (**d**) Experimental hysteresis curves of a Pd-AlO_x_-NiFe MTJ before and after molecular attachment. (**e**) Comparable simulation results for case (**b**) before and after molecule application. Inset shows the hysteresis graph in (**e**) for − 0.05 < B < 0.05. (**f**) Experimental hysteresis results for a TaCoNiFe/AlO_x_/NiFe MTJ before and after molecular attachment and (**g**) complementary simulation results for a FM-FM MTJ with and without attached molecules. Inset in (**g**) shows the hysteresis graph for − 0.075 < B < 0.075.

In this study, we utilize MTJMSD Heisenberg 3D model to investigate the magnetic hysteresis properties of paramagnetic molecule analog connected to three combinations of metal electrodes: ferromagnet-ferromagnet (FM-FM) (Fig. 1a), ferromagnet-paramagnet (FM-PM) (Fig. 1b), and ferromagnet-antiferromagnet (FM-AFM) (Fig. 1c). This paper mainly focuses on experimental studies that were conducted on pillar-shaped MTJMSD. We have conducted simulations to check if our MCS are producing reasonable results in light of experimental data. Experimental device fabrication and MCS methodology details have been published elsewhere^29^. We experimentally made Ta(2 nm)/Co(5 nm)/NiFe(5 nm)/AlO_x_(2 nm)/NiFe (10 nm) for the FM-FM-MTJ case. We fabricated Pd(10 nm)/AlO_x_ (2 nm)/NiFe (10 nm) for the paramagnet PM-FM-MTJ case. From this point onwards, MTJMSD with two ferromagnets (FM) electrodes, FM and paramagnetic (PM) metal electrode, and FM and antiferromagnetic(AFM) metal electrode are identified as FMFM-MTJMSD (Fig. 1a), FMPM-MTJMSD (Fig. 1b), FMAFM-MTJMSD(Fig. 1c), respectively. In the experimental studies, MTJMSD pillars were ≈5 µm diameter. We attached Octametallic molecular complex (OMC) across the insulating gap via the thiol terminal functional group^29^. OMC chemical structure included cyanide-bridged octametallic molecular cluster, [(pzTp)FeIII(CN)3]4[NiII(L)]4¬[O3SCF3]4 [(pzTp) = tetra(pyrazol-1-yl)borate; L = 1-S(acetyl)tris(pyrazolyl)decane]. The in-depth experimental^42,43^ and DFT-based theoretical^16^ details of OMCs and the method of chemically bonding OMCs across insulating gaps are published elsewhere^29^.

For the MCS study of MTJMSD, we have utilized Heisenberg atomic model^44^. The unitless energy of the MTJMSD atomic model was minimized using the metropolis algorithm via the Markov chain process^45^. The energy equation for the MTJMSD is shown in Eq. (1).

1 $$E=-{J}_{L}\left({\sum }_{i\in L}{\overrightarrow{S}}_{i}{\overrightarrow{S}}_{i+1}\right)-{J}_{R}\left({\sum }_{i\in R}{\overrightarrow{S}}_{i}{\overrightarrow{S}}_{i+1}\right)-{J}_{mL}\left({\sum }_{i\in L,i+1\in SMM}{\overrightarrow{S}}_{i}{\overrightarrow{S}}_{i+1}\right)-{J}_{mR}\left({\sum }_{i-1\in SMM,i\in R}{\overrightarrow{S}}_{i-1}{\overrightarrow{S}}_{i}\right)-{J}_{SMM}\left({\sum }_{i-1\in mol,i\in R}{\overrightarrow{S}}_{i-1}{\overrightarrow{S}}_{i}\right)-{D}_{SMM }\left({\sum }_{i\in L}{\overrightarrow{S}}_{i}^{2}\right)-{B}_{y}\left({\sum }_{i\in L}{\overrightarrow{S}}_{i}+{\sum }_{i\in R}{\overrightarrow{S}}_{i}+{\sum }_{i\in mol}{\overrightarrow{S}}_{i}\right).$$

In unitless Eq. (1) *S*_*i*_ represents atomic spins in the electrodes and spin of the molecule analog. All the exchange parameters in Eq. (1) are varied as unitless quantities. *J*_*L*_ and *J*_*R*_ represent the interatomic exchange coupling within the left and right electrodes, respectively. To set up the simulation model for FMFM-MTJMSD, both *J*_*L*_ and *J*_*R*_ was fixed to positive 1. For FMPM-MTJMSD *J*_*L*_ and *J*_*R*_ were fixed to 1 and 0, respectively. For FMAFM-MTJMSD *J*_*L*_ and *J*_*R*_ were assigned 1 and − 1, respectively. The SMM’s analog exchange coupling with left and right electrodes was determined by *J*_*mL*_ and *J*_*mR*_ , respectively. According to experimental and theoretical studies, the magnitude of *J*_*mL*_ and *J*_*mR*_ was ≈50% of the interatomic exchange coupling strength within electrodes^29^. Hence, considering that the comparable magnitude of molecule–metal coupling is analogous with respect to inter-atomic coupling in the metal electrodes, the magnitude of *J*_*mL*_ and *J*_*mR*_, was chosen to be 1 and − 1, respectively. The reason of selecting the opposite sign of molecule–metal electrode exchange coupling is due to the experimental study showing that OMC produced the opposite type of coupling with the two electrodes^29^.

In the present study, SMM is represented by simple atomic analog by utilizing the insights from related prior work. This approximation enabled us to set *J*_*SMM*_ (intramolecular coupling) and *D*_*SMM*_ (molecular anisotropy) to zero. Understandably, representing complex molecules with a generic paramagnetic atom with a net spin at high temperature is a significant simplification. However, we have shown that this approach has enabled us to estimate the magnitude of *J*_*mL*_ and *J*_*mR,*_ which is very close to the experimental results^29^. Interestingly, this simplification has also been successful in providing insights about the long-range impact on the cross junction-shaped MTJMSD^44^. In the powder form, OMC was experimentally shown to possess as high as S = 6 spin state below 10 K and stabilized into S = 2 spin state upon heating up to 60 K^42,43^. Due to the limitation of experimental techniques, we cannot find the exact spin state of those OMCs serving as spin channels between the ferromagnetic electrodes of MTJMSD in the experimental data of Fig. 1. However, from our prior work, we understand that molecule spin cannot be zero to yield the observed phenomenon^46^. We recently showed that molecule spin > 0.2 magnitude exhibits long-range ordering on the ferromagnetic electrodes. We also found that the nature of long-range molecule-induced order was similar for the molecule spin state over 0.2^46^. For this study at kT = 0.1 thermal energy, we chose a molecule spin state equal to 1 in light of our recent work. Our MCS study showed that for S = 0, magnetic electrodes of the MTJMSD were independent of each other, and no long-range ordering was observed as observed in the MFM and KPFM study at room temperature on MTJMSDs^46^.

In addition to our direct observations, other researchers’ work also provides evidence that any molecule directly connected to ferromagnetic electrodes undergoes dramatic changes. Recently, experimental studies showed that the hybridization of Cu-phenalenyl molecule and cobalt electrode energy states yields net magnetic moment on the molecules (with 1/2 spin state) near the interface at room temperature^47^. Spinterface, the interface between molecules and the magnetic electrodes, has started taking the form of a sub-branch in spintronics due to a spin-filtering-like phenomenon impacting the molecules and magnetic electrodes simultaneously^48^. Molecule–metal interaction impacted molecule and metal electrodes around the interface in a device type that included multiple molecule monolayers sandwiched between the two magnetic electrodes; molecules away from the interfaces were not impacted, similar to that along the interfaces^49^.

MTJMSD offers SMM a unique opportunity to develop strong hybridization with metallic electrodes because, unlike conventional devices, a robust insulator physically separates two metal electrodes to minimize interference from defects and unpredictable short circuits. MTJ-based molecular devices covalently connected each molecule of an array of 1D molecule chains between two ferromagnetic electrodes to yield unprecedented metamaterials and phenomena at room temperature^26^. In this state, SMM must assume a new electronic and magnetic form dictated by its hybridization with the electrodes that will be much different than the properties observed on isolated molecules. As discussed earlier in this paragraph, the molecule spin state becomes more robust in the connected state with the metal electrode. However, we have no knowledge about the *D*_*SMM*_ and *J*_*SMM*_ like parameters for the SMM connected to magnetic electrodes in our experimental study. Due to this reason, we have opted to use a generic paramagnetic atomic molecule analog that possesses a net spin irrespective of *D*_*SMM*_ and *J*_*SMM*_. Therefore, we fixed *D*_*SMM*_ and *J*_*SMM*_ to zero and assigned molecule spin to 1 spin state to molecule analog in the MCS. This approach has given insightful results in the context of experimental results^29,46,50^.

## Results and discussion

**Figure 2:**
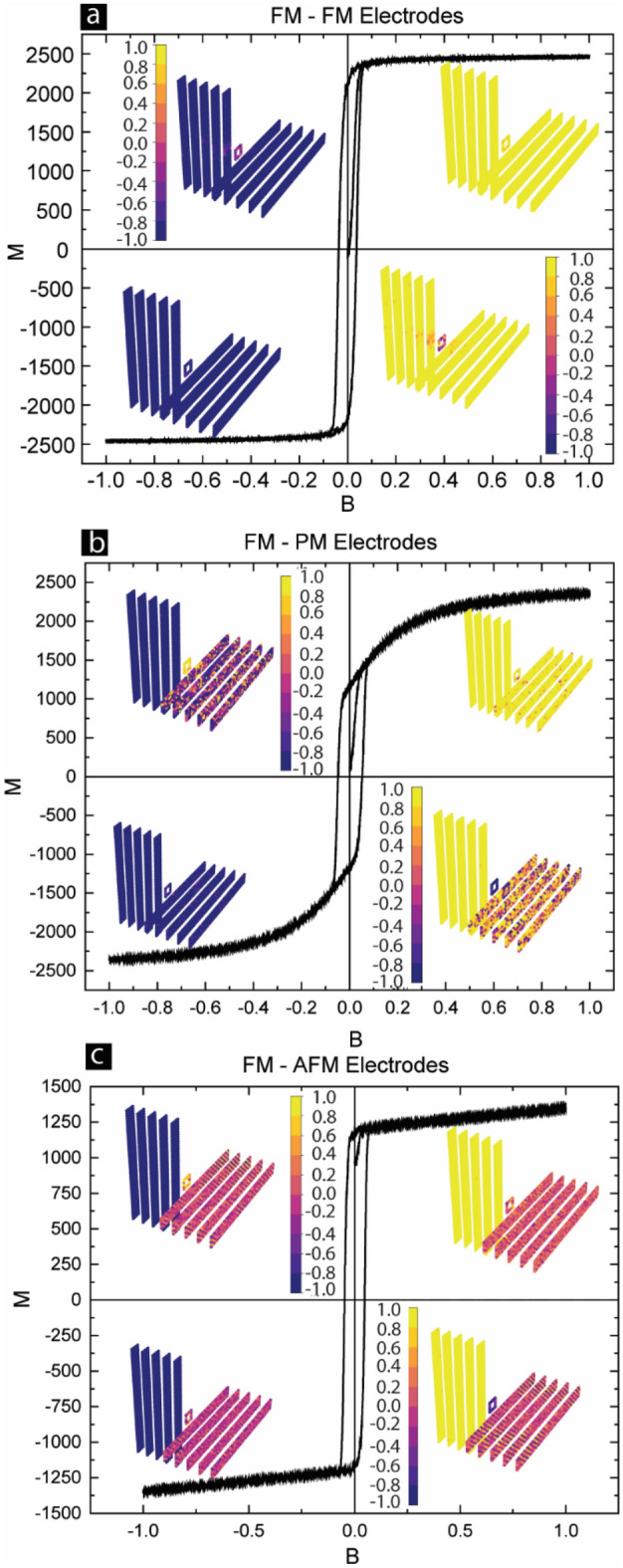
Monte Carlo simulation investigating the effect of varying magnetic field on MTJMSD devices designed with (**a**) FM/FM, (**b**) FM/PM, and (**c**) FM/AFM electrodes. The inset in each graph shows the snapshot of the stabilized device with the left electrode, molecule, and right electrode (clockwise starting at quadrant 1) in B = 1, 0.1, − 1, and – 0.1. The color bar shows the value of atomic spin for the device components.

To justify the validity of the MCS study with regard to experimental data, we simulated the 11 × 10 × 10 Heisenberg model. Since energy Eq. (1) is a unitless equation for the MTJMSD model, we used unitless parameters to represent atomic interactions such as* J*_*mL*_, *J*_*mR*_, *J*_*L*_ and *J*_*R*_. Variation of the magnitude of different unitless coupling energy parameters was relative to the inter-atomic bond strength of the electrode. MTJMSD’s electrodes were of 5 × 5 × 5 dimension. Molecules were placed at the perimeter of a 5 × 5 atomic square between the two electrodes along the edges. However, we studied the cross-junction-shaped Heisenberg model to investigate the molecule's long-range impact. Cross junction shaped MCS model included molecules along the perimeter of a 5 × 5 square at the crossing of 5 × 5 × 50 metal electrodes, as shown in Fig. 2 here and in the prior work^44,46^.

We simulated the magnetic hysteresis properties (M-H curve) by applying the in-plane magnetic field (B_y_) and measuring the magnetic moment. We ran 500 million iterations to attain a stable equilibrium state. The thermal energy of the simulation was set to 0.1 to keep the study relevant to the anticipated operating temperature around the room temperature. Here, we discuss the magnetic hysteresis curves for the different cases and elaborate on the evolution of molecules in association with the electrodes of MTJMSDs.

Figure 1 focuses on establishing the usefulness of MCS ability in producing trends similar to the experimental results obtained on pillar form MTJMSD. Our MCS cannot directly compare the magnitude of simulated data with the experimental data. It must be noted that the experimentally studied complex microscopic MTJMSD devices are several thousand times bigger than the atomic model size used in MCS. To study the representative MTJMSD toy model in MCS, we have followed the conventional algorithm for including a magnetic field in the MCS^45,51^. In the MCS, we focus on unitless energy minimization for different variations in magnetic fields. As per Eq. (1), the energy component associated with magnetic field variation is ${B}_{y}\left({\sum }_{i\in L}{\overrightarrow{S}}_{i}+{\sum }_{i\in R}{\overrightarrow{S}}_{i}+{\sum }_{i\in mol}{\overrightarrow{S}}_{i}\right)$. Since we vary magnetic field as a unitless parameter in the unitless energy equation, we have not used any unit for MCS-produced hysteresis graphs in this paper. Figure 1 only correlates MCS trends with experimental studies. Numerical values in the MCS study can be interpreted and understood relative to the electrode material defining parameters such as Heisenberg coupling strength within electrodes. Roughly, *J* = 1 Heisenberg Exchange coupling for the FM electrode corresponds to the Curie temperature (and associated thermal energy) for the ferromagnet used in the experimental studies.

Any molecular spintronics device will require extended metal electrodes to make molecule connections with the outside world. However, in this state, metal electrodes in and around the junction area will affect the MTJMSD switching ability among different states. We have a target to understand the response of molecules and the molecule–metal interfaces in cross-junction-shaped MTJMSD via the MCS. However, before discussing the MCS study of cross junctions, we have compared the MCS results for the pillar-shaped MTJMSD with experimentally tested ≈5 µm diameter MTJMSD. Magnetic hysteresis response before and after attaching OMC molecules on FMPM-MTJMSD is shown in Fig. 1d. OMC brought down the magnetic moment for the whole scan range of the in-plane magnetic field. We were unable to conduct the magnetic hysteresis study for a longer magnetic field range due to magnetometer limitation, and hence do not know what to expect for an extended magnetic field range. It is worth remembering that each metal electrode is a micron wide, ~ 10 nm thick, and possesses ~ 1 million atoms.

We compared the MCS-produced magnetic hysteresis loop for the FMPM-MTJ before and after hosting molecules to produce strong antiferromagnetic coupling between electrodes; each FM and PM electrode possessed 500 atoms (5 × 10 × 10). FMPM MTJ without molecules proceeds to saturation following a curved trend. It means switching between the initial to saturated state is not instantaneous and can be attributed to the presence of PM electrodes that generally follow a linear magnetization trend in the simulated MH curve. The MH data for FMPM-MTJ without molecule channels saturated around 0.25 magnetic field. Interestingly, the magnetic moment of the FMPM-MTJ, after hosting molecules, reached saturation around 0.7 magnetic field. It is apparent that molecule-impacted MTJ is harder to align as compared to MTJ without molecule. This MCS study provides an explanation for the experimental data in Fig. 1d. Due to the difference in saturation magnetic field before and after molecule involvement as a strong coupling agent, a decrease in magnetic moment occurred. MCS data in Fig. 1e also suggests that we had the ability to increase the magnetic field. Further, the magnetic moment will ultimately saturate at some higher field for the FMPM case (Fig. 1e). The inset in Fig. 1e also provides insights about the width of the hysteresis at zero magnetic moments in experimental results (Fig. 1d). We saw no measurable difference in width of hysteresis loop at zero magnetic moments on bare, and OMC treated experimental Pd/AlOx/NiFe tunnel junction (Fig. 1d). However, the width of hysteresis loop was smaller in MCS study after FMPM MTJ started hosting molecule (Fig. 1e). The reason is that number of atoms connected to molecules is 32, which is a significant number with respect 125 atoms in each metal electrode (≈25%). At the interfaces, FM and PM atoms have to align antiparallel due to molecule-based antiferromagnetic coupling. Due to this, molecule-enforced cancellation of magnetic moment occurred, leading to reduced hysteresis curve width (Inset of Fig. 1e). However, in the experimental study, the molecules only bridged a few thousand atoms in FM and PM electrodes possessing millions of atoms. Hence, molecule-impacted atoms in FM and PM electrodes were insignificant with respect to atoms in the electrodes. Thus, molecules make no difference in the width of the hysteresis loop (Fig. 1d).

We further investigated the hysteresis of FMFM MTJ with and without molecules experimentally (Fig. 1f) and theoretically (Fig. 1g). Experimental results showed that OMC transformed regular MTJ hysteresis loop into a linear magnetization response (Fig. 1f). Based on three independent magnetic measurements (Ferromagnetic resonance, Magnetic Force Microscopy, and SQUID magnetometry)^29^, we have concluded that OMC was able to produce strong antiferromagnetic coupling between the two ferromagnetic electrodes and produced a global antiparallel arrangement. Under an induced ≈5 µm wide antiparallel arrangement FM magnetic moment aligned antiparallel to each other and was stable for the whole magnetic field range of 5000 Oe. Due to the limitations of our magnetometer, we were unable to apply a magnetic field beyond 30,000 Oe (3 Tesla). The OMC-induced linear trend continued up to 30,000 Oe and went beyond the saturation level of bare MTJ. It is apparent that OMC has increased the saturation magnetic moment via the spin filtering process that has been discussed elsewhere^24^. Therefore, OMC has transformed FM electrodes into a highly spin-polarized state and then produced antiparallel alignment that could not be disturbed up to ≈50 °C above room temperature and under a strong magnetic field up to 3 T.

We simulated the pillar shape FMFM MTJ by MCS by assuming that FM electrodes are 100% spin-polarized^29^. In the MCS study, we applied strong antiferromagnetic coupling by introducing molecules along the side edges of the MTJ (Fig. 1g). We conducted an MCS simulation over a large magnetic field that is on the scale of coupling energy is comparable to interatomic exchange coupling within the electrodes. The hysteresis loop of FMFM MTJ became linear due to the molecule-induced exchange coupling (Fig. 1g) that is consistent with experimental observation for the FMFM MTJ case (Fig. 1f). However, since we have utilized 100% spin-polarized FM electrodes in the MCS, the linear curve arising due to molecular coupling finally saturate at the same level as achieved by the MTJ without molecular coupling (Fig. 1g). Unlike, experimental data where FM electrode was first transformed into low spin-polarized FM electrodes to high spin-polarized FM electrode in MCS study such situation do not arise. Inset shows that molecule-induced antiferromagnetic coupling produced a linear response until 0.1 fields. This field is around 10% of interatomic exchange coupling. It is obvious that we were unable to apply that strong field experimentally that will be equivalent to several tens of Tesla. An important point to note is that strong molecule induced linear magnetization response in MCS and was consistent with experimental results. Based on the consistency and strong correlation between experimental and MCS studies on pillar-shaped FMPM and FMFM MTJs, we validated the capability of MCS in simulating MTJMSD.

In order to make switchable devices with the ability to read and write, MTJMSD's different states pillar shape device structure is not suitable. Cross junction or cross-wire device architecture has been widely discussed and experimented with for harvesting the molecule attributes as the device element^52–56^. Hence, we explored crossbar-shaped MTJ where electrodes extend out in cross-junction form beyond the cross-section region (Insets of Fig. 2). Here, for the first time, we explore the hysteresis properties of FM-PM, FM-FM, and FM-AFM MTJMSDs (Fig. 2) to understand the impact of extended electrodes on the whole device.

FMFM-MTJMSD showed a typical hysteresis curve that resembles the hysteresis curve seen on FM electrodes in isolation (Fig. 2a). However, in the case of pillar-shaped FMFM-MTJMSD we experimentally (Fig. 1f) and theoretically (Fig. 1g) observed a linear MH curve. Comparing the two cases of pillar vs. cross junction shaped MTJMSD, we noted that extended FM electrodes beyond the junction area had dominated the MH graph. It is also noteworthy that in the initial stage of MH curve, when the magnetic field started increasing from zero, the MTJMSD magnetic moment was near zero (Fig. 2a). In the equilibrium state, two long FM electrodes were in the antiparallel state (Supplementary Material Fig. S1). The magnetic ordering on ferromagnetic electrodes was correlated with the molecule spin state (Supplementary Material Fig. S2). The length scale of molecule-induced magnetic ordering depended on the physical dimensions of the FM electrodes. In the supplementary materials, we show the paramagnetic molecule connected to 50-atom-long FM electrodes produced homogeneous magnetic ordering (Fig. S2a) as compared to the 200-atom-long FM electrode (Fig. S2b). It is clear that despite strong molecule coupling the FM electrodes dominate the MH curve.

In this case, when the starting state was in the antiparallel spin of two FM electrodes, the overall MH curve followed a linear path (Fig. 2a). The linear MH response from materials corresponds to paramagnetic or antiferromagnetic material. However, after reaching the saturation and subsequently reversing the magnetic field direction the equilibrium state-specific linear response could not be maintained. Rather, the MH curve appears as the FM electrode magnetization curve. It is noteworthy that for the pillar-shaped MTJMSD, where the electrode area was just the junction area, the MH curve followed a linear trend (Fig. 1g). Hence, we conclude that designing MTJMSD has to carefully consider the dimensions of the FM electrodes in seeking desired switchable attributes. We have also investigated the individual spin state of FM electrodes and molecules in the saturated and transition regimes of the MH curve and placed them as the inset (Fig. 2a). The spin vector is shown in the Y direction, that is, the direction of the magnetic field. In the saturation state, two FM electrodes and molecule spin vector are aligned in the same direction. However, at 0.1 field, the two electrodes are aligned in the parallel state, but the molecules' spin becomes highly disordered (Inset in quadrants 2 and 4 of Fig. 2a). Hence, molecules appear to be more sensitive than FM electrodes with regards to the magnetic field. Descending down from the saturation field when MTJMSD crosses zero fields, two magnetic electrodes are no longer antiparallel; otherwise, the magnetic moment should have been zero for zero fields. We expect FM electrodes to have multiple phases or domains around the transition point. In our prior experimental work, we have shown FM electrodes exhibited significantly different magnetic domains at the cross junction^28^. However, the main presence of magnetic domains is attributed to the presence of magnetic anisotropy. We have not included an anisotropy factor in the current study because of the reason that MTJMSD is the result of strong exchange coupling between two dissimilar ferromagnetic electrodes. Strong exchange coupling-induced magnetic anisotropy is less understood at this time, and we do not have a provision to incorporate it in the MCS study.

FMPM-MTJMSD with extended electrodes exhibited a similar magnetization response (Fig. 2b) as compared to pillar-shaped FMPM-MTJMSD (Fig. 1b,c). A hysteresis behavior was observable. Beyond the hysteresis zone, magnetization increased non-linearly and saturated (Fig. 2b). As shown in the inset 3D atomic structures of the simulated MTJMSDs, the PM electrode random spin orientation persisted for small magnetic field (Fig. 2b, quadrant 2 and 4). However, for the higher magnetic field PM electrode got aligned with the magnetic field and color contrast (Fig. 2b, quadrant 1 and quadrant 3). It is apparent that an increasing magnetic field will align two electrodes and molecules in the same direction, and this will correspond to the lowest resistance state. However, the molecule orientation is antiparallel to the FM electrode for the low magnetic field regime, and molecules are highly ordered (Fig. 2b, quadrants 2 and 4). In the low field state, FMPM MTJMSD will yield the highest resistance. A significant change in resistance is expected to occur when the magnetic field is switched from a low to a high state. It is likely that our MCS study is undermining the impact of thermal energy that may disrupt the spin ordering within molecules. If the molecule layer’s spin is disordered, then the transport will keep happening, and meager switching is expected between high and low magnetic fields.

Magnetization response for the case of FMAFM-MTJMSD also produced a characteristic hysteresis response for the low magnetic regime (Fig. 2c). As the magnetic field increased the magnetic moment increased linearly but never saturated or increased beyond 1300 count (Fig. 2c). However, in the FM-FM (Fig. 2a) and FM-PM (Fig. 2c) cases saturation state reached the magnetization magnitude of ≈2500. It is noteworthy that the antiferromagnetic ordering within the electrodes was strongly present in the low and high field regions (Fig. 2c, quadrant 1–4). This is due to the fact that inter-atomic exchange coupling between the nearest neighbors is as strong as it is in the FM electrode. This level of exchange coupling is beyond the capability of the external magnetic field. Interestingly, the molecules' spin states were rather compliant with the atom they bonded in the AFM electrode. Unlike the FM-PM case, where molecule order was dominated by the coupling with the FM electrode (Fig. 2a), in the FM-AFM case, molecule spin order was dominated by the coupling with the AFM electrode regime (Fig. 2c). Interestingly, in the high field regime molecules attained a rather independent state with respect to the two electrodes (Fig. 2c, quadrant 1 and 3). FM electrodes and molecules tend to be antiparallel with respect to each other (Fig. 2c, quadrant 4). Also, molecules attained uniform orientation with respect to the AFM electrode. We have not conducted experimental FM-AFM devices yet. It is expected that the unique situation in FMAFM-MTJMSD multiple resistance states may be materialized.

**Figure 3:**
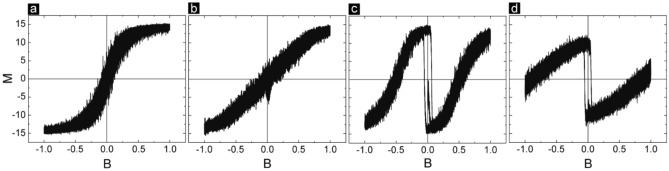
Monte Carlo simulation producing theoretical hysteresis loops for the (**a**) free paramagnetic molecule not attached to the MTJ, (**b**) molecules coupled to the FM-FM MTJ, (**c**) molecules coupled to the FM-PM MTJ, and (**d**) molecules coupled to the FM-AFM MTJ.

It is a matter of curiosity to know what happens to molecule spin state in the three forms of MTJMSDs as a function of the magnetic field. We have investigated a paramagnetic molecule analog's magnetic moment vs. magnetic field characteristics. If the molecule is not connected to any electrode, then it behaves like a paramagnetic entity; 16 molecules’ magnetic moment increases linearly between ± 0.2 field and saturates afterward (Fig. 3a). Interestingly, molecules behave much differently when bonded to the two FM electrodes of the FMFM-MTJMSD (Fig. 3b). In this case, molecule magnetic moment does not saturate until ± 1 (Fig. 3b). It is due to the fact that molecules are bonded to the atoms of FM electrodes and those FM electrode atoms are tied to the remaining FM electrodes atoms via the Heisenberg exchange coupling. Hence, in the FMFM-MTJMSD case, the magnetic field has to deal with a much bigger system. The linear response of molecule magnetic moment with magnetic field also suggests that FM electrode atoms near interfacial regions also cumulatively follow linear magnetic moment vs. magnetic field response. The molecular response in FMPM MTJMSD was intriguingly different. There were two paramagnetic transitions and one hysteresis loop in the center (Fig. 3c). As the magnetic field started increasing in the positive direction, the molecule’s magnetic moment steeply decreased from 0 to − 14. This is because molecules form strong antiferromagnetic coupling with the FM electrode. As the magnetic field organizes FM electrodes and PM electrodes, the minimum energy state will correspond to the case when 16 molecules align antiparallel to the FM electrode and parallel to the PM electrode interfacial atoms. As the magnetic field increases beyond 0.5, the magnetic moments of the molecules start stabilizing ~ 90 degrees with respect to the direction of the applied field. As the magnetic field reaches the maximum limit, the molecule’s magnetic moment completely flips to be in the direction FM electrode (Fig. 3c). At this point, molecules FM and PM electrodes are all in the direction (Fig. 2b, quadrant 1). It is noteworthy that when FM electrodes and molecules follow opposite directions due to antiferromagnetic exchange coupling between the FM electrode and molecule. When the magnetic field starts to decrease, the molecule traverses the same path. However, at zero magnetic field, the moment of the FM electrode is finite and heading to be in a negative direction. As soon as FM electrode switches from + to – magnetic field region, molecules quickly switch to the opposite state with respect to FM electrodes (Fig. 3c). As a result, the molecule magnetic moment becomes positive for the negative field and keeps decreasing to the other end of the saturation state. i.e. − 14. Very intriguingly, molecule response in the FMAFM MTJMSD case was somewhat similar to FMPM case near the low magnetic region. The mechanism is also quite the same; since the molecule makes antiferromagnetic coupling with the FM electrode, the molecule cohort has to align in the opposite direction with respect to the FM electrode (Fig. 3d). However, as the magnetic field grows stronger molecules start aligning with respect to the AFM electrode. Since AFM electrodes consist of a series of atoms that are antiparallel to each other, the molecule at the interface will be connected to the half-spin-up and half-spin-down AFM atoms. Hence, the total sum of the 16 molecules will be close to zero because ~ 8 molecules are up and 8 molecules are down due to the influence of AFM electrodes (Fig. 3d). It is not clear to us why the molecule is influenced by FM electrode for the low magnetic field range and by the AFM electrode for the high magnetic field range. It is quite clear that a molecule spin state will lose identity when connected to different combinations of electrodes.

**Figure 4:**
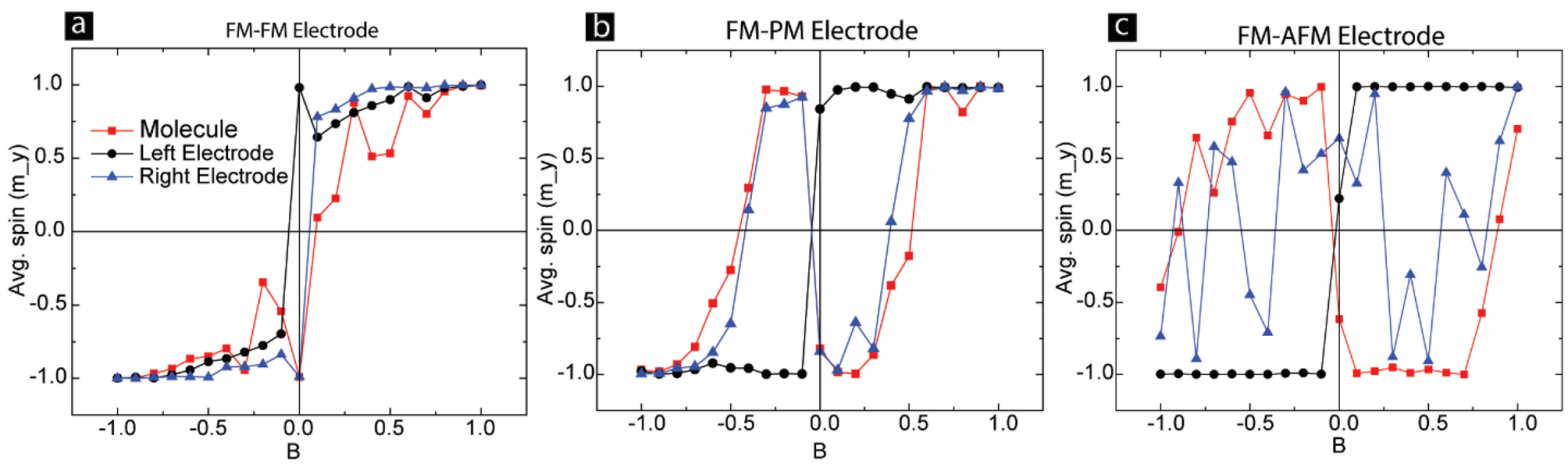
Monte Carlo simulation giving the average spin of the molecule and it’s nearest neighbors in the left and right electrode, in the direction of the applied magnetic field. The left electrode in all cases is a FM and the right electrode is a (**a**) FM, (**b**) PM, and (**c**) AFM representing different cases of MTJMSD.

After investigating molecule response in different devices with diverse electrode configurations, we investigated the molecule and interface spin properties along the magnetic field direction. However, for this step, we calculated the average spin of molecules and the average spins of two electrodes directly connected to the molecules. We first investigated FMFM-MTJ (Fig. 4a). For the zero magnetic fields, the spins of the two FM electrodes were opposite each other (Fig. 4a). It is critically important information suggesting that in the equilibrium state, a whole cross junction that is ten times more in length as compared to the junction length of 5 atoms possessed antiparallel alignment. As the magnetic field increased, the two electrodes started to align in the same direction. Also, it is noteworthy that molecules and two metal electrodes head to saturation levels at different rates. Molecules were the slowest to respond and saturate (Fig. 4a). With increasing magnetic field, both FM electrodes’ interfaces aligned in the same direction. The molecule was initially parallel and antiparallel with respect to the left and right FM electrodes' interfacial atoms, respectively. Molecule spin along the field direction switched from low to high state at the non-zero magnetic field. From the practical application perspective, the application of a magnetic field can switch the 100% spin-polarized FM electrodes from an antiparallel to a parallel state, and this switching may produce a large resistance change in the transport properties (Fig. 4a).

Interestingly, the FMPM-MTJ case right electrode and molecule followed the same trajectory (Fig. 4b). At the PM-molecule interface, the PM electrode atoms made direct antiferromagnetic exchange bonds with the molecules and no coupling with other PM electrode atoms. As a result, PM interface atoms settle opposite to the molecules' magnetic moment (Fig. 4b). Interestingly, FM electrode atoms at the interface follow the molecule magnetic moment because of the ferromagnetic coupling between FM electrode and molecules. Remarkably, FM and PM electrode atoms behave the same way but in the opposite direction up to ≈0.6 magnetic fields and are finally saturated in the same direction. The molecule and FM electrode atoms at the interface started moving in the direction of PM-molecule interface atoms near 0.25 magnetic field magnitude. It is important to note that the switching field for FM-PM cases is 50–75% less than the exchange coupling strength.

Contrary to our hypothesis that strong ordering in AFM electrode may lead to stabilized magnetic moments of the whole system at the interface, we found a quite chaotic situation (Fig. 4c). In the FM-AFM case, molecules under the influence of molecule-induced antiferromagnetic coupling in the interface area follow a very different course (Fig. 4c) as compared to the whole device (Fig. 2c). Most striking part is that molecule magnetic direction and the right AFM electrode are fluctuating with respect to the field direction. The right AFM electrode, where atoms are expected to be antiparallel to each other, appears to be, in fact, aligned in different directions other than the Y direction. It is apparent that the magnetic moment of the AFM electrode atoms at the interface does not saturate to unity (Fig. 4c). The large fluctuations in interfacial AFM electrode atoms are closely followed by the molecule’s magnetic moment. Molecules appear to be aligned antiparallel to the direction of the magnetic field in general. Interestingly, molecules have the same coupling strength with the FM electrode do not align in the direction of the FM electrode. Molecules appear to strike a dynamic equilibrium under strong coupling with FM and AFM electrodes. As the magnetic field strength increases to 1, which is equivalent to interatomic bonding and molecule coupling strength, molecules and AFM electrodes tend to align in the direction of FM electrode. We anticipate that such a magnetic field, relative to interatomic electrode bond energy, may be difficult to achieve. It is important to understand a vast range of possibilities and device attributes are expected for the cases when molecule coupling with the two electrodes is dissimilar and is not very strong.

## Conclusions

In this study, we showed the following key points:

1. The dimensions of the MTJMSD matter. A pillar-shaped MTJMSD retains a perfectly antiparallel state with two FM electrodes. However, MTJMSD magnetic hysteresis with extended electrodes is dominated by the electrodes. Interestingly, at the interfaces, a strong molecule-induced exchange coupling dominates the magnetic alignment of two FM electrodes. In practical application, a large switch in transport is expected when a small magnetic field is applied. FM electrodes appear to have different magnetic orientations as compared to the electrode segments away from the junction areas.
2. The strength and nature of the coupling between electrodes and molecules decide the relative orientations of magnetic molecules with respect to two electrodes. Hence, a molecule loses its identity when connected to two metal electrodes. The success of the future simulation and modeling of the experimentally studied devices must have provisions to accurately map the molecule coupling with the electrodes to yield realistic results.
3. This study utilized 100% spin-polarized FM electrodes due to the experimental observations showing that magnetic molecule coupling can transform a regular FM electrode into nearly 100% spin-polarized electrodes.
4. The advantage of using AFM metal electrodes to yield clean device properties may not be possible if the molecule induces strong exchange coupling with the two electrodes.
5. Future theoretical studies may benefit from combining the strength of DFT, Micromagnetic Simulations, and MCS for mapping and comprehending the equilibrium and dynamic properties of MTJMSDs.
6. MCS studies suggest that the molecule analog's magnetic moment is influenced by the FM electrode for the low magnetic field range and by the AFM electrode for the high magnetic field range.
7. At the interface, FM electrodes were opposite to each other along the direction of magnetic fields, even though in a cumulative hysteresis loop for MTJMSD, two electrodes aligned in the same direction.

### Supplementary Information

Supplementary Figures.

## Data Availability

Data included in this paper is available upon reasonable request. Dr. Pawan Tyagi, corresponding author, should be contacted for requesting data.
