# Supplementary material for: Magnetic molecules lose identity when connected to different combinations of magnetic metal electrodes in MTJ-based molecular spintronics devices (MTJMSD)
Source: Sci Rep. 2023 Sep 27;13:16201. doi: 10.1038/s41598-023-42731-9 (PMC10533507; doi:10.1038/s41598-023-42731-9)
Supplement: Supplementary file 1 — Supplementary Figures. [file 41598_2023_42731_MOESM1_ESM.docx]

# **Supplementary Information**

**Magnetic Molecules Lose Identity When Connected to Different Combinations of Magnetic Metal Electrodes in MTJ-Based Molecular Spintronics Devices (MTJMSD)**

Eva Mutunga^1^, Christopher D’Angelo^1^, Pawan Tyagi^1*^

Center for Nanotechnology Research and Education, Mechanical Engineering, 4200 Connecticut Avenue, University of the District of Columbia, Washington DC 2008, USA^1^


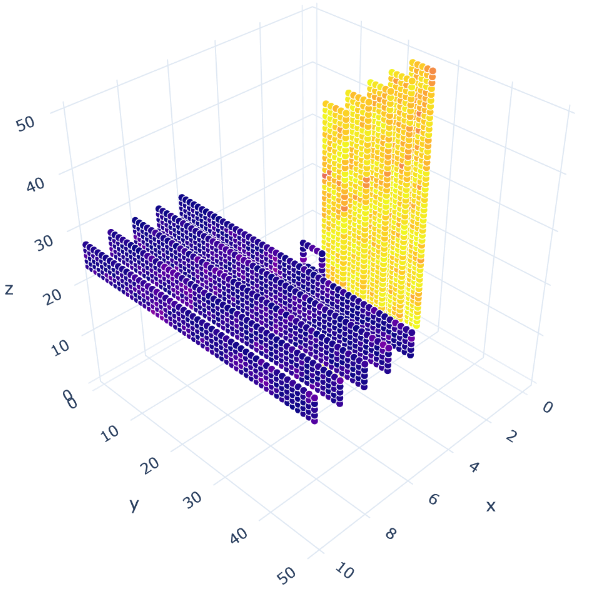

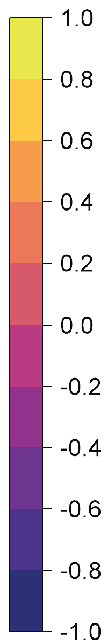


Figure S1: MCS simulation producing 3D view of the stabilized MTJMSD Heisenberg Model after the completion of a Monte Carlo simulation. Solid yellow and blue colors represent spin-up(1) and spin-down (-1) states. Color identification between the yellow and blue represent different orientation.

The impact range of the molecule was calculated by computing the correlation factor between the molecule's Spin state and the ferromagnetic electrode of 50 and 200 atom length. The correlation between molecule spin and the magnetic electrode's individual atoms was computed by calculating the dot product of the molecule spin vector and each FM electrode atom. More details about the computation of correlated phase calculation have been published elsewhere, as mentioned in the main body of the manuscript.


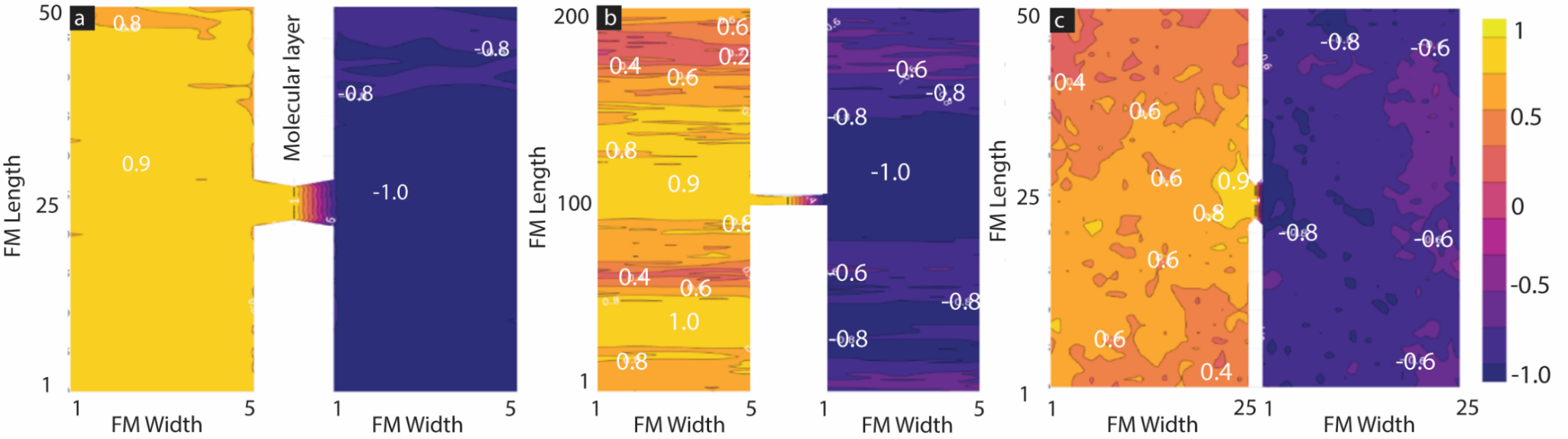

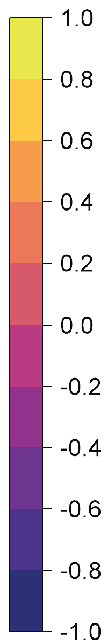


Figure S2: 2D view of molecule spin correlated magnetic electrodes' spin spatial phase distribution for FM electrode with (a) 50 atom length (b) and 200 atom length. Solid yellow (1) and blue colors represent positive correlation and negative correlation states with reference to molecules. The yellow and blue colors represent different correlation magnitude ranges between 1 and -1.
